# Effects of a botanical feed additive blend of capsicum oleoresin, clove and garlic essential oils on growth performance and fecal dry matter in nursery pigs

**DOI:** 10.1093/tas/txaf016

**Published:** 2025-02-05

**Authors:** Ty H Kim, Jason C Woodworth, Mike D Tokach, Joel M DeRouchey, Robert D Goodband, Jordan T Gebhardt, Mark T Knauer, Christiaan P A van de Ligt, Emma H Wall

**Affiliations:** Department of Animal Sciences and Industry, College of Agriculture, Kansas State University, Manhattan, KS 66506-0201, USA; Department of Animal Sciences and Industry, College of Agriculture, Kansas State University, Manhattan, KS 66506-0201, USA; Department of Animal Sciences and Industry, College of Agriculture, Kansas State University, Manhattan, KS 66506-0201, USA; Department of Animal Sciences and Industry, College of Agriculture, Kansas State University, Manhattan, KS 66506-0201, USA; Department of Animal Sciences and Industry, College of Agriculture, Kansas State University, Manhattan, KS 66506-0201, USA; Department of Diagnostic Medicine/Pathobiology, College of Veterinary Medicine, Kansas State University, Manhattan, KS 66506-0201, USA; Department of Animal Science, College of Agriculture and Life Sciences, North Carolina State University, Raleigh, NC 27695, USA; Selko USA, Indianapolis, IN, USA; Nutreco Exploration, Nutreco, The Netherlands

**Keywords:** botanicals, copper, essential oils, fecal dry matter, nursery pigs, zinc

## Abstract

Two experiments were conducted to determine the effects of a botanical-derived feed additive containing capsicum oleoresin, clove and garlic essential oils (CCG; Fytera Start, Selko, Indianapolis, IN) in nursery pigs fed with or without pharmacological levels of Zn and Cu. In Exp. 1756 pigs (Duroc × Landrace/Large White composite (Smithfield Premium Genetics), initially 7.8 ± 0.09 kg) were used in a 40-d study to determine the effects of CCG level on growth performance of nursery pigs fed pharmacological levels of Zn and Cu. In Exp. 2340 barrows (DNA 200 × 400, initially 6.1 ± 0.08 kg) were used in 38-d study to determine the effect of CCG in diets with or without pharmacological levels of Zn and Cu on growth performance and fecal dry matter (DM). For both experiments, pigs were randomly allotted to pens which were allotted to 1 of 4 dietary treatments in a completely randomized design. There were 9 pigs per pen and 21 pens per treatment in Exp. 1 and 5 pigs per pen and 17 pens per treatment in Exp. 2. Dietary treatments in Exp. 1 were corn-soybean meal based with pharmacological levels of Zn and Cu and included either 0, 25, 50, or 100 mg/kg of CCG. Dietary treatments in Exp. 2 were arranged in a 2 × 2 factorial with main effects of CCG (none or 100 mg/kg) and nutritional or pharmacological levels of Zn and Cu. All Exp. 2 diets contained 110 mg/kg of Zn and 16.5 mg/kg of Cu from the trace mineral premix. For both experiments, pharmacological levels of Zn were added at 3,000 and 2,000 mg/kg in phase 1 and 2, respectively and Cu was added at 250 mg/kg in all phases. For Exp. 1, overall average daily gain (ADG) increased (linear, *P *< 0.05) and average daily feed intake (ADFI) tended to increase (linear, *P *< 0.10) as CCG increased from 0 to 100 mg/kg. For Exp. 2, there was a CCG × Zn/Cu interaction observed for overall ADG and ADFI (*P *< 0.05) where CCG numerically increased ADG and ADFI in pigs fed nutritional levels of Zn and Cu; but reduced ADG and ADFI in pigs fed pharmacological levels of Zn and Cu. There was a Zn/Cu × day interaction (*P *= 0.001) for fecal DM, in which there was no difference (*P *> 0.10) in fecal DM on d 10, but pigs fed pharmacological levels of Zn and Cu had lower (*P *< 0.001) fecal DM on d 21 compared to pigs fed nutritional levels of Zn and Cu. In summary, when included in diets with pharmacological levels of Zn and Cu, feeding increasing levels of CCG increased ADG in Exp. 1 but did not improve performance in Exp 2.

## Teaser

Feeding increasing levels of a blend of capsicum oleoresin, clove and garlic essential oils increased average daily gain and tended to increase average daily feed intake in diets containing pharmacological levels of Zn and Cu in one experiment but not in another.

## INTRODUCTION

Weaning is a stressful event in a pig’s life and is often marked by poor performance and increased incidence of post-weaning diarrhea (Pluske et al., 1997). Adding pharmacological levels of Zn and Cu in nursery pig diets is a common strategy to improve growth performance and fecal consistency of nursery pigs (Hill et al., 2000; Bikker et al., 2016). The effects of pharmacological levels of Zn and Cu are well established and appear to be the result of their effect on gut morphology and antimicrobial activity (Højberg et al., 2005; Liu et al., 2018). Because the addition of pharmacological levels of Zn and Cu are being limited for environmental reasons, there is considerable research being conducted to evaluate alternatives.

Phytogenics are plant-derived feed additives that act through molecular gut physiology to decrease inflammation and improve gut integrity and function (Franz et al., 2010; Omonijo et al., 2017). However, due to differences in the composition of phytogenics, their effects on pig performance can vary (Franz et al., 2010). In literature reviews, Franz et al. (2010) and Zeng et al. (2015) summarized the results of numerous studies using capsicum oleoresin, clove, and garlic compounds (either independently or in combination with other plant compounds) and reported equivocal effects on growth performance. Zeng et al. (2015) reported three studies with capsicum oleoresin in combination with carvacrol and cinnamaldehyde, with two observing positive effects and one observing negative effects on growth performance. Zeng et al. (2015) also reported three studies with clove in combination with other compounds, and all observed a positive response on growth performance. Recently a blend of botanical-derived active molecules comprised of capsicum oleoresin, clove and garlic essential oils (CCG; Fytera Start, Selko, Indianapolis, IN) has been introduced for use in nursery pig diets. Previous research has shown decreased frequency of diarrhea and improved intestinal morphology when feeding CCG in pigs challenged with *E. coli* (Wong et al., 2022). However, no data is available to determine the effects of this CCG blend in diets with pharmacological levels of Zn and Cu. Our objective for Exp. 1 was to evaluate the effects of increasing levels of this blend of phytogenic compounds in pigs fed diets containing pharmacological levels of Zn and Cu on weanling pig growth performance. Our next objective (Exp. 2) was to use this ideal dosage from Exp. 1 and determine the effects on growth performance and fecal dry matter (DM) of nursery pigs fed diets with or without pharmacological levels of Zn and Cu. Our hypothesis was that this blend of phytogenic compounds could potentially show additive responses with pharmacological levels of Zn and Cu.

## MATERIALS AND METHODS

### Experiment 1

The study was conducted at the North Carolina Department of Agriculture Tidewater Research Station in Plymouth, NC. Animal care and use standards were followed as set forth by the Guide for Care and Use of Agricultural Animals in Research and Teaching.

A total of 756 pigs (Duroc sires × Landrace/Large White composite females; Smithfield Premium Genetics, Rose Hill, NC), initially 7.8 ± 0.09 kg, were used in a 40-d growth study. Pigs were weaned at 23 d of age, randomly allotted to pens based on initial body weight (BW) and then pens were allotted to 1 of 4 dietary treatments in a completely randomized design. There were 9 pigs per pen and 21 pens per treatment. Each pen (1.45 × 1.45 m) had metal tri-bar floors and contained a dry self-feeder and a nipple drinker for ad libitum access to feed and water.

The four dietary treatments were corn-soybean meal based with pharmacological levels of Zn and Cu and included either 0, 25, 50, or 100 mg/kg CCG in all phases (Table 1). Zinc was added at 3,000, 2,000, and 110 mg/kg in phases 1, 2, and 3, respectively. Copper was added at 250 mg/kg in all 3 phases. To achieve pharmacological levels of Zn and Cu, Zn from ZnO and Cu from tribasic copper chloride were added to the basal diets. Diets were fed in three phases from d 0 to 10, d 10 to 20, and d 20 to 40. Phase 1 and 2 diets were manufactured at the North Carolina State University feed mill in pellet form, and phase 3 diets were manufactured at the North Carolina State University Tidewater feed mill in meal form.

**Table 1.** Ingredient composition of Exp. 1 diets (as-fed basis)^1^

Ingredient, %	Phase 1	Phase 2	Phase 3
Corn	49.73	55.76	68.03
Soybean meal	23.25	26.80	28.70
Base mix 1^2^	25.00	15.00	----
Base mix 2^3^	1.75	2.25	3.25
Zinc oxide	0.25	0.17	----
Tribasic copper chloride^4^	0.02	0.02	0.02
CCG^5^	+/-	+/-	+/-
Total	100	100	100
Calculated analysis			
CP, %	20.6	20.5	19.0
Lys, %	1.53	1.52	1.44
Ca, %	0.69	0.67	0.68
P, %	0.58	0.56	0.53
Zn, mg/kg	3,000	2,000	110
Cu, mg/kg	250	250	250

^1^Phase 1 diets were fed from d 0 to 10. Phase 2 diets were fed from d 10 to 20. Phase 3 diets were fed from d 20 to 40.

^2^Contained 18.84% CP, 1.75% Lys, 1.40% Ca, 0.80% P, 4,562 mg/kg Zn, 291 mg/kg Cu, and 2,205 FTU of phytase/kg.

^3^Contained 17.92% CP, 10.30% Lys, 18.00% Ca, 4.70% P, 3,053 mg/kg Zn, 4,692 mg/kg Cu, and 15,102 FTU of phytase/kg.

^4^IntelliBond C II, 54% Cu.

^5^Fytera Start (a blend of capsicum oleoresin, clove and garlic essential oils; Selko, Indianapolis, IN) was added at 0, 25, 50, or 100 mg/kg of the diet.

Individual pigs were weighed and feed disappearance was recorded on d 0, 10, 20, and 40 to determine average daily gain (ADG), average daily feed intake (ADFI), and feed efficiency (G:F).

### Experiment 2

The protocol used in this experiment was approved by the Kansas State University Institutional Animal Care and Use Committee (4506.12). The study was conducted at the Kansas State University Segregated Early Weaning facility in Manhattan, KS. The facility has two identical barns that are completely enclosed, environmentally controlled, and mechanically ventilated. Each pen (1.22 × 1.22 m) had metal tri-bar floors and contained a 4-hole, dry self-feeder and a cup waterer for ad libitum access to feed and water.

A total of 340 barrows (DNA 200 × 400, initially 6.1 ± 0.08 kg) were used in a 38-d growth study. Pigs were weaned at approximately 21 d of age, randomly allotted to pens based on initial BW, and then pens were allotted to 1 of 4 dietary treatments in a completely randomized design. There were 5 pigs per pen and 17 pens per treatment across two barns (8 replicate pens in one barn and 9 replicate pens in the other).

Treatments were arranged in a 2 × 2 factorial with main effects of CCG (none or 100 mg/kg, based on results of Exp. 1) and nutritional or pharmacological levels of Zn and Cu. All diets contained 110 mg/kg of Zn (ZnSO_4_) and 16.5 mg/kg of Cu (CuSO_4_) from the trace mineral premix as the Nutritional levels. Pharmacological levels were 3,000 and 2,000 mg/kg of Zn in phase 1 and 2, respectively, and 250 mg/kg of Cu in all three phases. To achieve expected levels of Zn and Cu in the diet, Zn from ZnO and Cu from CuSO_4_ were added. The experimental diets were manufactured at the Kansas State University O.H. Kruse Feed Technology Innovation Center in Manhattan, KS (Table 2). Phase 1 diets were fed in pellet form, and phase 2 and 3 diets were fed in meal form. Complete diet samples were taken during bagging of experimental diets from every fourth bag using a feed probe, pooled into one homogenized sample per dietary treatment, and stored at -20°C until analysis. Complete diet samples were ground to reduce particle size and submitted for Zn and Cu analysis (Method 985.01; AOAC International, 2000; Cumberland Valley Analytical Services, Waynesboro, PA). Analysed Zn and Cu levels for all three phases were similar to the calculated values initially used in formulation (Table 3).

**Table 2.** Ingredient composition of Exp. 2 diets (as-fed basis)^1^

Ingredient, % Zn/Cu^2^:	Phase 1		Phase 2		Phase 3	
	Nutr.	Pharm.	Nutr.	Pharm.	Nutr.	Pharm.
Corn	45.29	44.76	57.57	57.18	65.75	65.65
Soybean meal	15.60	15.63	23.32	23.34	30.52	30.53
Fish meal	4.50	4.50	----	----	----	----
Whey powder	25.00	25.00	10.00	10.00	----	----
Enzymatically treated soybean meal^3^	5.00	5.00	5.00	5.00	----	----
Soybean oil	2.00	2.00	----	----	----	----
Limestone	0.25	0.25	0.83	0.83	0.81	0.81
Monocalcium P	0.65	0.65	1.18	1.18	0.83	0.83
Sodium chloride	0.30	0.30	0.55	0.55	0.60	0.60
L-Lys-HCl	0.40	0.40	0.50	0.50	0.48	0.48
DL-Met	0.21	0.21	0.22	0.22	0.20	0.20
L-Thr	0.18	0.18	0.22	0.22	0.21	0.21
L-Trp	0.04	0.04	0.04	0.04	0.04	0.04
L-Val	0.13	0.13	0.14	0.14	0.12	0.12
Trace mineral premix	0.15	0.15	0.15	0.15	0.15	0.15
Vitamin premix	0.25	0.25	0.25	0.25	0.25	0.25
Phytase^4^	0.06	0.06	0.06	0.06	0.06	0.06
Zinc oxide	----	0.40	----	0.26	----	----
Copper sulfate	----	0.09	----	0.09	----	0.09
CCG^5^	+/-	+/-	+/-	+/-	+/-	+/-
Total	100	100	100	100	100	100
Calculated analysis						
CP, %	20.5	20.4	20.7	20.7	20.8	20.8
Ca, %	0.71	0.71	0.77	0.77	0.65	0.65
STTD P, %	0.63	0.63	0.56	0.56	0.44	0.44
Ca:P	1.00	1.00	1.14	1.14	1.14	1.14
Zn, mg/kg	110	3,000	110	2,000	110	110
Cu, mg/kg	16.5	250	16.5	250	16.5	250

^1^Phase 1 diets were fed from d 0 to 10. Phase 2 diets were fed from d 10 to 21. Phase 3 diets were fed from d 21 to 38.

^2^Nutritional levels of Zn and Cu were 110 and 16.5 mg/kg, respectively, for all three phases. Pharmacological levels of Zn were 3,000 and 2,000 mg/kg in phase 1 and 2, respectively, and pharmacological levels of Cu were 250 mg/kg in all three phases.

^3^HP300; Hamlet Protein; Findlay, OH.

^4^Ronozyme HiPhos 2,700 (DSM, Parsippany, NJ) included at 1,486 FYT/kg provided an estimated release of 0.12% STTD P.

^5^Fytera Start (a blend of capsicum oleoresin, clove and garlic essential oils; Selko, Indianapolis, IN) was added at 100 mg/kg to the diets.

**Table 3.** Analysed composition of Exp. 2 diets (as-fed basis)^1^

Zn/Cu^2^:	Nutritional		Pharmacological	
Item, % CCG^3^:	No	Yes	No	Yes
Phase 1				
Zn, mg/kg	149	154	3,103	2,597
Cu, mg/kg	23	22	250	258
Phase 2				
Zn, mg/kg	141	143	2,069	1,990
Cu, mg/kg	21	24	266	269
Phase 3				
Zn, mg/kg	139	164	160	128
Cu, mg/kg	26	33	253	228

^1^Complete diet samples were taken during bagging of experimental diets from every fourth bag, pooled into one homogenized sample per dietary treatment and analyzed for Zn and Cu (Method 985.01; AOAC International, 2000) at Cumberland Valley Analytical Services, Waynesboro, PA.

^2^Zinc from ZnO and copper from CuSO_4_.

^3^Fytera Start (a blend of capsicum oleoresin, clove and garlic essential oils; Selko, Indianapolis, IN) was added at 100 mg/kg to the diets.

Individual pigs were weighed and feed disappearance was recorded on d 0, 10, 17, 21, 31, and 38 to determine ADG, ADFI, and G:F. On d 10 and 21 of the experiment, fecal samples were collected from the same three randomly selected pigs in each pen and analyzed for fecal DM. After collection, fecal samples were dried at 55°C in a forced air oven for 48 h, and the ratio of dried to wet fecal weight determined its DM. Fecal samples were maintained separately for each pig and the average of the three samples from each pen was then used for statistical analysis.

### Statistical Analysis

#### Experiment 1.

Experimental data were analyzed as a completely randomized design with pen as the experimental unit using the lmer package of R (version 4.2.2 (2022-10-31), R Foundation for Statistical Computing, Vienna, Austria; R Core Team, 2022). Treatment served as a fixed effect, and room served as a random effect. Linear and quadratic contrasts were constructed with increasing CCG levels. Differences were considered significant at *P *≤ 0.05 and marginally significant at 0.05 < *P *≤ 0.10.

#### Experiment 2.

Growth performance data were analyzed as a completely randomized design with pen serving as the experimental unit. Bodyweight at d 0 was used as a covariate for all responses except d 0 BW. The main effects of CCG and Zn/Cu and their interactions were tested. Fecal DM data were analyzed as a completely randomized design with pen as the experimental unit with the fixed effects of day, treatment, and the associated interaction accounting for repeated measures over time. Growth and fecal DM data were analyzed using the lmer package of R (version 4.2.2 (2022-10-31), R Foundation for Statistical Computing, Vienna, Austria; R Core Team, 2022). Differences were considered significant at *P *≤ 0.05 and marginally significant at 0.05 < *P *≤ 0.10.

## RESULTS

### Experiment 1

From d 0 to 10, there was no effect of increasing CCG on ADG (Table 4). Average daily feed intake tended (quadratic, *P *= 0.085) to decrease as CCG increased from 0 to 50 mg/kg but then increased from 50 to 100 mg/kg CCG. As a result, G:F tended (quadratic, *P *= 0.074) to increase from 0 to 50 mg/kg CCG but then returned to control values when fed at 100 mg/kg. From d 10 to 20, the G:F response (quadratic, *P *= 0.093) was similar as that observed from d 0 to 10. Increasing CCG had no effect on ADG or ADFI. From d 0 to 20, there was no effect on ADG or ADFI, but like observed from d 0 to 10, G:F increased from 0 to 50 mg/kg CCG but then returned to control values (quadratic, *P* < 0.036) at 100 mg/kg CCG. From d 20 to 40, ADG increased (linear, *P *= 0.024) as CCG increased. There was no effect on ADFI or G:F. Overall from d 0 to 40, ADG increased (linear, *P *= 0.005) and ADFI tended to increase (linear, *P *= 0.061) as CCG increased, with no effect on G:F.

**Table 4.** Effect of increasing botanical blend on growth performance of nursery pigs fed pharmacological levels of zinc and copper (Exp. 1)^1^

Item	CCG^2^, mg/kg				SEM	*P* =	
	0	25	50	100		Linear	Quadratic
BW, kg							
d 0	7.8	7.8	7.8	7.8	0.09	0.527	0.398
d 10	10.1	10.0	10.1	10.1	0.08	0.319	0.570
d 20	15.6	15.6	15.8	15.8	0.14	0.225	0.844
d 40	28.7	28.3	28.8	29.4	0.40	0.012	0.168
d 0 to 10 (Phase 1)							
ADG, g	209	208	213	219	6.5	0.213	0.833
ADFI, g	291	283	281	300	8.1	0.285	0.085
G:F, g/kg	726	742	767	736	27.8	0.642	0.074
d 10 to 20 (Phase 2)							
ADG, g	581	585	601	593	10.3	0.265	0.266
ADFI, g	857	854	862	881	15.9	0.119	0.583
G:F, g/kg	680	686	699	676	12.5	0.743	0.093
d 0 to 20 (Phases 1 and 2)							
ADG, g	386	387	399	397	5.8	0.124	0.503
ADFI, g	558	553	557	575	11.8	0.109	0.263
G:F, g/kg	693	701	717	693	13.3	0.989	0.036
d 20 to 40 (Phase 3)							
ADG, g	652	635	652	680	21.4	0.024	0.144
ADFI, g	1,179	1,144	1,177	1,207	29.9	0.114	0.219
G:F, g/kg	553	556	555	565	15.0	0.287	0.755
d 0 to 40 (Overall)							
ADG, g	518	510	525	537	11.0	0.005	0.360
ADFI, g	866	847	867	889	16.4	0.061	0.212
G:F, g/kg	599	604	607	607	13.1	0.409	0.529

^1^A total of 756 pigs (Duroc × Landrace/Large White composite (Smithfield Premium Genetics, Rose Hill, NC); initially 7.8 ± 0.09 kg BW) were used in a 40-d growth study with 9 pigs per pen and 21 replications per treatment.

^2^Capsicum oleoresin, clove and garlic essential oils (Fytera Start; Selko, Indianapolis, IN).

### Experiment 2

From d 0 to 10, there were no CCG × Zn/Cu interactions observed nor main effect of CCG (Table 5). However, pigs fed pharmacological levels of Zn and Cu had increased (*P *< 0.001) ADG, ADFI, G:F, and d 10 BW compared to pigs fed nutritional levels of Zn and Cu. From d 10 to 21 and d 0 to 21, there was a CCG × Zn/Cu interaction (*P *< 0.05) observed for ADG, ADFI, and d 21 BW where the addition of CCG resulted in a numerical increase in ADG and ADFI in pigs fed nutritional levels of Zn and Cu; however, in pigs fed pharmacological levels of Zn and Cu, the addition of CCG resulted in a numerical decrease in ADG and ADFI. From d 10 to 21 pharmacological levels of Zn and Cu increased G:F (*P *= 0.033) compared to pigs fed nutritional levels of Zn and Cu.

**Table 5.** Interactive effect of a botanical blend and zinc and copper supplementation on nursery pig growth performance and fecal dry matter (Exp. 2)^1^

Zn/Cu^2^:	Nutritional		Pharmacological		SEM	*P* =		
Item^3^ CCG^4^:	No	Yes	No	Yes		CCG × Zn/Cu	CCG	Zn/Cu
BW, kg								
d 0	6.1	6.1	6.0	6.1	0.08	0.545	0.008	0.608
d 10	6.8	6.9	7.3	7.2	0.16	0.242	0.932	< 0.001
d 21	11.1^b^	11.6^b^	13.0^a^	12.5^a^	0.26	0.007	0.886	< 0.001
d 38	21.2^c^	22.1^bc^	24.0^a^	22.9^ab^	0.35	0.002	0.811	< 0.001
d 0 to 10 (Phase 1)								
ADG, g	71	81	125	115	15.6	0.242	0.932	< 0.001
ADFI, g	107	112	142	131	15.9	0.184	0.630	< 0.001
G:F, g/kg	638	695	861	857	44.4	0.438	0.556	< 0.001
d 10 to 21 (Phase 2)								
ADG, g	394^b^	425^b^	511^a^	477^a^	13.4	0.008	0.922	< 0.001
ADFI, g	463^b^	506^b^	624^a^	582^a^	17.0	0.006	0.993	< 0.001
G:F, g/kg	853	842	823	820	13.4	0.725	0.645	0.033
d 0 to 21 (Phases 1 and 2)								
ADG, g	240^b^	261^b^	324^a^	305^a^	15.5	0.032	0.888	< 0.001
ADFI, g	293^b^	318^b^	391^a^	367^a^	15.5	0.019	0.942	< 0.001
G:F, g/kg	818	819	832	829	11.7	0.861	0.937	0.248
d 21 to 38 (Phase 3)								
ADG, g	591^b^	617^ab^	645^a^	615^ab^	11.2	0.006	0.909	0.011
ADFI, g	829	860	884	869	16.2	0.114	0.632	0.029
G:F, g/kg	713	719	730	709	8.4	0.064	0.351	0.600
d 0 to 38 (Overall)								
ADG, g	397^c^	419^bc^	467^a^	443^ab^	9.4	0.007	0.892	< 0.001
ADFI, g	533^c^	559^bc^	610^a^	592^ab^	13.6	0.048	0.780	< 0.001
G:F, g/kg	745	750	766	750	6.8	0.083	0.450	0.087
Fecal DM^5^, %								
d 10	25.6	23.8	25.1	25.5	0.93	0.154	0.323	0.398
d 21	24.1	23.8	20.5	21.7	0.93	0.271	0.578	< 0.001

^1^A total of 340 barrows (DNA 200 × 400, initially 6.1 ± 0.08 kg BW) were used in a 38-d growth trial with 5 pigs per pen and 17 replications per treatment.

^2^Zinc from ZnO was included in the diet at 110 or 3,000 mg/kg in phase 1,110 or 2,000 mg/kg in phase 2, and 110 mg/kg in phase 3 and copper from CuSO_4_ was included in the diet at 16.5 or 250 mg/kg throughout the 38-d study for the nutritional and pharmacological treatments, respectively.

^3^BW d 0 was used as a covariate for all responses except BW d 0.

^4^Capsicum oleoresin, clove and garlic essential oils (Fytera Start; Selko, Indianapolis, IN) included in the diet at either 0 or 100 mg/kg throughout the 38-d study.

^5^CCG × Zn × Day, *P* = 0.817; CCG × Zn/Cu, *P* = 0.075; CCG × Day, *P* = 0.275; Zn/Cu × Day, *P* = 0.001; CCG, *P* = 0.758; Zn/Cu, *P* = 0.026; Day, *P* < 0.001.

From d 21 to 38, there was a CCG × Zn/Cu interaction (*P *< 0.05) observed for ADG and d 38 BW in which the addition of CCG numerically increased ADG in pigs fed nutritional levels of Zn and Cu; however, in pigs fed pharmacological levels of Zn and Cu, the addition of CCG resulted in a numerical decrease in ADG. Average daily feed intake was increased (*P *= 0.029) in pigs fed pharmacological additions of Zn and Cu compared to those fed nutritional levels with no effect of CCG observed. There was a tendency for a CCG × Zn/Cu interaction (*P *= 0.064) observed for G:F in which the addition of CCG numerically increased G:F in pigs fed nutritional levels of Zn and Cu but numerically decreased G:F in pigs fed pharmacological levels of Zn and Cu.

From d 0 to 38 (overall), there was a CCG × Zn/Cu interaction (*P *< 0.05) observed for ADG and ADFI in which the addition of CCG numerically increased ADG and ADFI in pigs fed nutritional levels of Zn and Cu; however, when pigs were fed pharmacological levels of Zn and Cu, the addition of CCG numerically decreased ADG and ADFI. There was a tendency for a CCG × Zn/Cu interaction (*P *= 0.083) observed for G:F in which the addition of CCG had no effect on G:F when pigs were fed nutritional levels of Zn and Cu, but the addition of CCG numerically decreased G:F in pigs fed pharmacological levels of Zn and Cu.

For fecal DM, there was a Zn/Cu × day interaction (*P *= 0.001) in which there was no difference in fecal DM regardless of Zn and Cu level on d 10, but pigs fed pharmacological levels of Zn and Cu had lower fecal DM (*P *< 0.001) compared to those fed nutritional levels of Zn and Cu on d 21. There was also a tendency for a CCG × Zn/Cu interaction (*P *= 0.075) in which the addition of CCG numerically decreased fecal DM in pigs fed nutritional levels of Zn and Cu, but the addition of CCG numerically increased fecal DM in pigs fed pharmacological levels of Zn and Cu. Fecal DM was greater (*P *< 0.001) on d 10 compared to d 21.

## DISCUSSION

Zinc and Cu are trace minerals required in growing pigs at 50 to 100 mg/kg and 3 to 6 mg/kg, respectively (NRC, 2012). However, including high levels, often referred to as pharmacological levels, of Zn from ZnO and Cu from CuSO_4_ or other Zn or Cu sources in nursery pig diets is a common practice in the U.S. to reduce post-weaning diarrhea and promote growth (Hill et al., 2000; Bikker et al., 2016). According to Liu et al. (2018), the positive effect of pharmacological levels of Zn on growth and fecal consistency is due to the mineral’s effect on gut morphology and integrity and anti-inflammatory activity. When fed at pharmacological levels, Zn and Cu have also been observed to have anti-microbial effects (Højberg et al., 2005; Liu et al., 2018).

The independent effects of pharmacological levels of Zn and Cu are well documented. Shelton et al. (2011) observed increased ADG, ADFI, and final BW in pigs fed 3,000 and 2,000 mg/kg Zn from ZnO in the first 28 d after weaning compared to pigs fed nutritional levels of Zn. Similarly, Pérez et al. (2011) observed increased overall nursery ADG and ADFI in pigs fed 250 mg/kg Cu from CuSO_4_ compared to pigs fed nutritional levels of Cu. Hill et al. (2000) observed firmer fecal scores in pigs fed 3,000 mg/kg Zn from ZnO or 250 mg/kg Cu from CuSO_4_ compared to pigs fed nutritional levels of Zn or Cu. Similar to the previous literature, in Exp. 2, pigs fed pharmacological levels of Zn and Cu had increased ADG, ADFI, and BW throughout the overall nursery period compared to pigs fed nutritional levels of Zn and Cu.

Phytogenic compounds, or botanicals, are a relatively new class of feed additives with increasing research interest, and they can include herbs, spices, essential oils, and oleoresins that are derived from the specialized metabolites found in plants (Windisch et al., 2008). The potential modes of action of plant derived feed additives include decreased inflammation, improved intestinal health, antimicrobial activity, antioxidative effects, and improved diet palatability (Franz et al., 2010; Omonijo et al., 2017). However, phytogenic feed additives can vary based on plant and geographical origin, harvesting season, and processing technique (Windisch et al., 2008). Phytogenic feed additives can be derived from a single type of plant compound or a blend of different plant compounds. As a result of the variation in composition, botanical feed additives can have a wide range of growth promoting, immunostimulatory, and antimicrobial benefits which can lead to variable results (Turner et al., 2001).

The botanical additive used in the current studies is a blend of capsicum oleoresin, clove and garlic essential oils. Biggs et al. (2020) observed increased G:F with the addition of 0.01% capsicum oleoresin in diets without pharmacological levels of Zn or Cu when fed to pigs in thermoneutral and heat stress environments. Long et al. (2021) observed increased ADG, apparent total tract digestibility of nutrients, and antioxidant capacity in pigs fed 1.6 mg/kg capsicum oleoresin compared to pigs fed a control diet or one with 75 mg/kg chlortetracycline. Yan et al. (2011) observed increased ADG and ADFI in finishing pigs fed 0.2% garlic powder compared to those fed a diet without garlic powder. However, there were no benefits in growth performance in pigs fed 0.4% garlic powder. In nursery pigs, Huang et al. (2011) observed linear and quadratic increases ADG, ADFI, and G:F as garlic powder increased from 0% to 0.025% of the diet. Mohammadi et al. (2014) observed increased ADG and G:F in broiler chickens fed 300 mg/kg of clove essential oil compared to those not fed the essential oil.

Liu et al. (2013) determined the effects of separately including 10 mg/kg of three different plant extracts (PE) including capsicum oleoresin, garlic botanical, or turmeric oleoresin in diets without pharmacological levels of Zn and Cu fed to pigs either infected or not infected with *Escherichia coli.* Although the authors did not include the exact blend of botanical compounds, two of their compounds, capsicum oleoresin and garlic, were evaluated herein. Liu et al. (2013) observed increased ADG in the early nursery period in pigs fed any of the PE compared to the control diet when not infected with *E. coli.*, but there was no difference in overall performance. Pigs fed any of the PE had firmer feces compared to pigs fed the control diet regardless of *E. coli.* challenge. When challenged with *E. coli* F18, Wong et al. (2022) observed that pigs fed 100 mg/kg CCG in diets without pharmacological levels of Zn or Cu had numerically greater ADG and G:F and reduced frequency of diarrhea compared to pigs not fed CCG.

There is little research on the interactive effects of phytogenics with other growth-promoting agents, such as pharmacological Zn and Cu. Feldpausch et al. (2018) observed a Cu × essential oil (EO) interaction where providing 125 mg/kg added Cu resulted in a numeric increase in G:F compared to pigs fed nutritional levels of Cu when EO was not present in the diet; however, when EO was included in the diet, pigs fed 125 mg/kg added Cu had a numeric decrease in G:F compared to pigs fed nutritional levels of Cu. In Exp. 2, there was a CCG × Zn/Cu interaction on ADG and ADFI in which the addition of CCG numerically increased ADG and ADFI in pigs fed nutritional levels of Zn and Cu; however, in pigs fed pharmacological levels of Zn and Cu, the inclusion of CCG numerically decreased ADG and ADFI.

In summary, in Exp. 1 increasing levels of a blend of capsicum oleoresin, clove and garlic essential oils increased overall ADG and tended to increase ADFI. In Exp. 2, the blend of phytogenic compounds numerically increased ADG and ADFI in pigs fed nutritional levels of Zn and Cu but numerically decreased ADG and ADFI in pigs fed pharmacological levels of Zn and Cu. There was no impact of the botanical blend or Zn and Cu level on fecal DM on d 10. Feeding pharmacological levels of Zn and Cu increased ADG and ADFI but decreased fecal DM on d 21. In conclusion, feeding increasing levels of CCG increased ADG in Exp. 1, but this response was not observed when CCG was fed in combination with pharmacological levels of Zn and Cu in Exp 2.
